# Supplementary material for: Pyruvate Dehydrogenase Kinase 1 inhibition mediated oxidative phosphorylation enhancement in cartilage promotes osteoarthritis progression
Source: BMC Musculoskelet Disord. 2023 Jul 20;24:597. doi: 10.1186/s12891-023-06585-6 (PMC10357736; doi:10.1186/s12891-023-06585-6)
Supplement: Supplementary file 1 — Additional file 1. [file 12891_2023_6585_MOESM1_ESM.zip › BP.Gsea.1653623667859/GOBP_NUCLEAR_EXPORT.html]

Details for gene set GOBP\_NUCLEAR\_EXPORT[GSEA]

|  || Dataset | 55457\_collapsed\_to\_symbols.cls.cls #OA\_versus\_normal.cls.cls #OA\_versus\_normal\_repos |
| Phenotype | cls.cls#OA\_versus\_normal\_repos |
| Upregulated in class | normal |
| GeneSet | GOBP\_NUCLEAR\_EXPORT |
| Enrichment Score (ES) | -0.4088631 |
| Normalized Enrichment Score (NES) | -1.8692503 |
| Nominal p-value | 0.0 |
| FDR q-value | 0.06413035 |
| FWER p-Value | 0.832 |
Table: GSEA Results Summary

  

Fig 1: Enrichment plot: GOBP\_NUCLEAR\_EXPORT      
 Profile of the Running ES Score & Positions of GeneSet Members on the Rank Ordered List

  

| SYMBOL | TITLE | RANK IN GENE LIST | RANK METRIC SCORE | RUNNING ES | CORE ENRICHMENT || 1 | NMD3 | NMD3 ribosome export adaptor [Source:HGNC Symbol;Acc:HGNC:24250] | 240 | 0.791 | -0.0001 | No |
| 2 | HHEX | hematopoietically expressed homeobox [Source:HGNC Symbol;Acc:HGNC:4901] | 270 | 0.774 | 0.0158 | No |
| 3 | XPO7 | exportin 7 [Source:HGNC Symbol;Acc:HGNC:14108] | 443 | 0.686 | 0.0186 | No |
| 4 | RITA1 | RBPJ interacting and tubulin associated 1 [Source:HGNC Symbol;Acc:HGNC:25925] | 753 | 0.580 | 0.0080 | No |
| 5 | PCID2 | PCI domain containing 2 [Source:HGNC Symbol;Acc:HGNC:25653] | 1376 | 0.447 | -0.0301 | No |
| 6 | PARK7 | Parkinsonism associated deglycase [Source:HGNC Symbol;Acc:HGNC:16369] | 1406 | 0.442 | -0.0219 | No |
| 7 | IFI27 | interferon alpha inducible protein 27 [Source:HGNC Symbol;Acc:HGNC:5397] | 1627 | 0.409 | -0.0295 | No |
| 8 | GAS6 | growth arrest specific 6 [Source:HGNC Symbol;Acc:HGNC:4168] | 1942 | 0.368 | -0.0454 | No |
| 9 | NUP133 | nucleoporin 133 [Source:HGNC Symbol;Acc:HGNC:18016] | 1951 | 0.367 | -0.0374 | No |
| 10 | THOC7 | THO complex 7 [Source:HGNC Symbol;Acc:HGNC:29874] | 2170 | 0.341 | -0.0464 | No |
| 11 | XPO1 | exportin 1 [Source:HGNC Symbol;Acc:HGNC:12825] | 2477 | 0.305 | -0.0632 | No |
| 12 | TGFB1 | transforming growth factor beta 1 [Source:HGNC Symbol;Acc:HGNC:11766] | 2490 | 0.303 | -0.0569 | No |
| 13 | POM121L2 | POM121 transmembrane nucleoporin like 2 [Source:HGNC Symbol;Acc:HGNC:13973] | 2841 | 0.266 | -0.0781 | No |
| 14 | ATXN1 | ataxin 1 [Source:HGNC Symbol;Acc:HGNC:10548] | 2862 | 0.264 | -0.0734 | No |
| 15 | POLDIP3 | DNA polymerase delta interacting protein 3 [Source:HGNC Symbol;Acc:HGNC:23782] | 2869 | 0.264 | -0.0677 | No |
| 16 | BARD1 | BRCA1 associated RING domain 1 [Source:HGNC Symbol;Acc:HGNC:952] | 3051 | 0.245 | -0.0761 | No |
| 17 | NUP93 | nucleoporin 93 [Source:HGNC Symbol;Acc:HGNC:28958] | 3194 | 0.231 | -0.0817 | No |
| 18 | STRADA | STE20 related adaptor alpha [Source:HGNC Symbol;Acc:HGNC:30172] | 3210 | 0.229 | -0.0775 | No |
| 19 | CDK5 | cyclin dependent kinase 5 [Source:HGNC Symbol;Acc:HGNC:1774] | 3400 | 0.211 | -0.0873 | No |
| 20 | EMD | emerin [Source:HGNC Symbol;Acc:HGNC:3331] | 3404 | 0.211 | -0.0826 | No |
| 21 | ENY2 | ENY2 transcription and export complex 2 subunit [Source:HGNC Symbol;Acc:HGNC:24449] | 3561 | 0.197 | -0.0901 | No |
| 22 | RIOK2 | RIO kinase 2 [Source:HGNC Symbol;Acc:HGNC:18999] | 3615 | 0.192 | -0.0897 | No |
| 23 | KHDRBS1 | "KH RNA binding domain containing, signal transduction associated 1 [Source:HGNC Symbol;Acc:HGNC:18116]" | 3748 | 0.182 | -0.0958 | No |
| 24 | NUP155 | nucleoporin 155 [Source:HGNC Symbol;Acc:HGNC:8063] | 3824 | 0.174 | -0.0976 | No |
| 25 | SDAD1 | SDA1 domain containing 1 [Source:HGNC Symbol;Acc:HGNC:25537] | 4229 | 0.134 | -0.1260 | No |
| 26 | UPF1 | UPF1 RNA helicase and ATPase [Source:HGNC Symbol;Acc:HGNC:9962] | 4434 | 0.118 | -0.1392 | No |
| 27 | NUP42 | nucleoporin 42 [Source:HGNC Symbol;Acc:HGNC:17010] | 4479 | 0.115 | -0.1399 | No |
| 28 | DDX19B | DEAD-box helicase 19B [Source:HGNC Symbol;Acc:HGNC:2742] | 4500 | 0.113 | -0.1388 | No |
| 29 | NUP85 | nucleoporin 85 [Source:HGNC Symbol;Acc:HGNC:8734] | 4630 | 0.101 | -0.1465 | No |
| 30 | RAE1 | ribonucleic acid export 1 [Source:HGNC Symbol;Acc:HGNC:9828] | 4697 | 0.096 | -0.1494 | No |
| 31 | NRDE2 | "NRDE-2, necessary for RNA interference, domain containing [Source:HGNC Symbol;Acc:HGNC:20186]" | 5019 | 0.071 | -0.1729 | No |
| 32 | TCF7L2 | transcription factor 7 like 2 [Source:HGNC Symbol;Acc:HGNC:11641] | 5034 | 0.070 | -0.1723 | No |
| 33 | EIF4ENIF1 | eukaryotic translation initiation factor 4E nuclear import factor 1 [Source:HGNC Symbol;Acc:HGNC:16687] | 5330 | 0.045 | -0.1944 | No |
| 34 | NXF3 | nuclear RNA export factor 3 [Source:HGNC Symbol;Acc:HGNC:8073] | 5648 | 0.020 | -0.2187 | No |
| 35 | AHCYL1 | adenosylhomocysteinase like 1 [Source:HGNC Symbol;Acc:HGNC:344] | 5837 | 0.006 | -0.2333 | No |
| 36 | SP100 | SP100 nuclear antigen [Source:HGNC Symbol;Acc:HGNC:11206] | 5899 | 0.002 | -0.2380 | No |
| 37 | TPR | "translocated promoter region, nuclear basket protein [Source:HGNC Symbol;Acc:HGNC:12017]" | 5919 | 0.001 | -0.2395 | No |
| 38 | CCHCR1 | coiled-coil alpha-helical rod protein 1 [Source:HGNC Symbol;Acc:HGNC:13930] | 5954 | -0.002 | -0.2421 | No |
| 39 | NUP88 | nucleoporin 88 [Source:HGNC Symbol;Acc:HGNC:8067] | 5976 | -0.003 | -0.2437 | No |
| 40 | FRAT1 | FRAT regulator of WNT signaling pathway 1 [Source:HGNC Symbol;Acc:HGNC:3944] | 6027 | -0.007 | -0.2474 | No |
| 41 | GLE1 | GLE1 RNA export mediator [Source:HGNC Symbol;Acc:HGNC:4315] | 6058 | -0.009 | -0.2496 | No |
| 42 | EIF6 | eukaryotic translation initiation factor 6 [Source:HGNC Symbol;Acc:HGNC:6159] | 6140 | -0.015 | -0.2555 | No |
| 43 | SMG6 | SMG6 nonsense mediated mRNA decay factor [Source:HGNC Symbol;Acc:HGNC:17809] | 6175 | -0.019 | -0.2578 | No |
| 44 | NUTF2 | nuclear transport factor 2 [Source:HGNC Symbol;Acc:HGNC:13722] | 6536 | -0.045 | -0.2849 | No |
| 45 | WASHC4 | WASH complex subunit 4 [Source:HGNC Symbol;Acc:HGNC:29174] | 6612 | -0.050 | -0.2896 | No |
| 46 | NUP62 | nucleoporin 62 [Source:HGNC Symbol;Acc:HGNC:8066] | 6800 | -0.063 | -0.3027 | No |
| 47 | RBM15B | RNA binding motif protein 15B [Source:HGNC Symbol;Acc:HGNC:24303] | 6867 | -0.069 | -0.3063 | No |
| 48 | SETD2 | "SET domain containing 2, histone lysine methyltransferase [Source:HGNC Symbol;Acc:HGNC:18420]" | 6926 | -0.074 | -0.3091 | No |
| 49 | RANBP3 | RAN binding protein 3 [Source:HGNC Symbol;Acc:HGNC:9850] | 7043 | -0.083 | -0.3162 | No |
| 50 | CHTOP | chromatin target of PRMT1 [Source:HGNC Symbol;Acc:HGNC:24511] | 7184 | -0.095 | -0.3249 | No |
| 51 | RANBP17 | RAN binding protein 17 [Source:HGNC Symbol;Acc:HGNC:14428] | 7194 | -0.096 | -0.3234 | No |
| 52 | CALR | calreticulin [Source:HGNC Symbol;Acc:HGNC:1455] | 7291 | -0.102 | -0.3285 | No |
| 53 | NUP62CL | nucleoporin 62 C-terminal like [Source:HGNC Symbol;Acc:HGNC:25960] | 7601 | -0.127 | -0.3497 | No |
| 54 | POLR2D | RNA polymerase II subunit D [Source:HGNC Symbol;Acc:HGNC:9191] | 7726 | -0.136 | -0.3562 | No |
| 55 | NPAP1 | nuclear pore associated protein 1 [Source:HGNC Symbol;Acc:HGNC:1190] | 7737 | -0.136 | -0.3537 | No |
| 56 | CAMK1 | calcium/calmodulin dependent protein kinase I [Source:HGNC Symbol;Acc:HGNC:1459] | 7795 | -0.141 | -0.3549 | No |
| 57 | CPSF6 | cleavage and polyadenylation specific factor 6 [Source:HGNC Symbol;Acc:HGNC:13871] | 7885 | -0.147 | -0.3584 | No |
| 58 | MCM3AP | minichromosome maintenance complex component 3 associated protein [Source:HGNC Symbol;Acc:HGNC:6946] | 8077 | -0.161 | -0.3695 | No |
| 59 | NOL6 | nucleolar protein 6 [Source:HGNC Symbol;Acc:HGNC:19910] | 8081 | -0.161 | -0.3660 | No |
| 60 | SFN | stratifin [Source:HGNC Symbol;Acc:HGNC:10773] | 8118 | -0.164 | -0.3649 | No |
| 61 | MALT1 | MALT1 paracaspase [Source:HGNC Symbol;Acc:HGNC:6819] | 8120 | -0.164 | -0.3611 | No |
| 62 | PPM1A | "protein phosphatase, Mg2+/Mn2+ dependent 1A [Source:HGNC Symbol;Acc:HGNC:9275]" | 8125 | -0.165 | -0.3575 | No |
| 63 | NUP214 | nucleoporin 214 [Source:HGNC Symbol;Acc:HGNC:8064] | 8301 | -0.178 | -0.3670 | No |
| 64 | NCBP2 | nuclear cap binding protein subunit 2 [Source:HGNC Symbol;Acc:HGNC:7659] | 8478 | -0.192 | -0.3763 | No |
| 65 | THOC5 | THO complex 5 [Source:HGNC Symbol;Acc:HGNC:19074] | 8549 | -0.198 | -0.3771 | No |
| 66 | HNRNPA2B1 | heterogeneous nuclear ribonucleoprotein A2/B1 [Source:HGNC Symbol;Acc:HGNC:5033] | 8576 | -0.200 | -0.3744 | No |
| 67 | SIRT7 | sirtuin 7 [Source:HGNC Symbol;Acc:HGNC:14935] | 8733 | -0.211 | -0.3817 | No |
| 68 | SUPT6H | "SPT6 homolog, histone chaperone and transcription elongation factor [Source:HGNC Symbol;Acc:HGNC:11470]" | 8752 | -0.213 | -0.3781 | No |
| 69 | PKD1 | "polycystin 1, transient receptor potential channel interacting [Source:HGNC Symbol;Acc:HGNC:9008]" | 8755 | -0.213 | -0.3732 | No |
| 70 | DDX25 | DEAD-box helicase 25 [Source:HGNC Symbol;Acc:HGNC:18698] | 8815 | -0.218 | -0.3727 | No |
| 71 | CSE1L | chromosome segregation 1 like [Source:HGNC Symbol;Acc:HGNC:2431] | 9000 | -0.231 | -0.3816 | No |
| 72 | NCBP1 | nuclear cap binding protein subunit 1 [Source:HGNC Symbol;Acc:HGNC:7658] | 9149 | -0.244 | -0.3874 | No |
| 73 | HSPA9 | heat shock protein family A (Hsp70) member 9 [Source:HGNC Symbol;Acc:HGNC:5244] | 9173 | -0.246 | -0.3834 | No |
| 74 | NUP107 | nucleoporin 107 [Source:HGNC Symbol;Acc:HGNC:29914] | 9274 | -0.253 | -0.3853 | No |
| 75 | RAN | "RAN, member RAS oncogene family [Source:HGNC Symbol;Acc:HGNC:9846]" | 9478 | -0.272 | -0.3948 | No |
| 76 | TXN | thioredoxin [Source:HGNC Symbol;Acc:HGNC:12435] | 9487 | -0.273 | -0.3890 | No |
| 77 | NXF2 | nuclear RNA export factor 2 [Source:HGNC Symbol;Acc:HGNC:8072] | 9577 | -0.281 | -0.3893 | No |
| 78 | XPOT | exportin for tRNA [Source:HGNC Symbol;Acc:HGNC:12826] | 9597 | -0.283 | -0.3841 | No |
| 79 | ABCE1 | ATP binding cassette subfamily E member 1 [Source:HGNC Symbol;Acc:HGNC:69] | 9675 | -0.289 | -0.3833 | No |
| 80 | XPO6 | exportin 6 [Source:HGNC Symbol;Acc:HGNC:19733] | 9759 | -0.297 | -0.3828 | No |
| 81 | NXT1 | nuclear transport factor 2 like export factor 1 [Source:HGNC Symbol;Acc:HGNC:15913] | 9983 | -0.319 | -0.3927 | No |
| 82 | SMG5 | SMG5 nonsense mediated mRNA decay factor [Source:HGNC Symbol;Acc:HGNC:24644] | 10190 | -0.340 | -0.4009 | Yes |
| 83 | SRSF3 | serine and arginine rich splicing factor 3 [Source:HGNC Symbol;Acc:HGNC:10785] | 10280 | -0.349 | -0.3996 | Yes |
| 84 | RANGAP1 | Ran GTPase activating protein 1 [Source:HGNC Symbol;Acc:HGNC:9854] | 10288 | -0.349 | -0.3919 | Yes |
| 85 | ANP32B | acidic nuclear phosphoprotein 32 family member B [Source:HGNC Symbol;Acc:HGNC:16677] | 10327 | -0.353 | -0.3865 | Yes |
| 86 | RBM22 | RNA binding motif protein 22 [Source:HGNC Symbol;Acc:HGNC:25503] | 10345 | -0.355 | -0.3795 | Yes |
| 87 | PRKACA | protein kinase cAMP-activated catalytic subunit alpha [Source:HGNC Symbol;Acc:HGNC:9380] | 10611 | -0.384 | -0.3912 | Yes |
| 88 | DHX9 | DExH-box helicase 9 [Source:HGNC Symbol;Acc:HGNC:2750] | 10627 | -0.386 | -0.3832 | Yes |
| 89 | YTHDC1 | YTH domain containing 1 [Source:HGNC Symbol;Acc:HGNC:30626] | 10687 | -0.391 | -0.3786 | Yes |
| 90 | SMG7 | SMG7 nonsense mediated mRNA decay factor [Source:HGNC Symbol;Acc:HGNC:16792] | 10877 | -0.414 | -0.3837 | Yes |
| 91 | XPO4 | exportin 4 [Source:HGNC Symbol;Acc:HGNC:17796] | 10912 | -0.419 | -0.3765 | Yes |
| 92 | CHP1 | calcineurin like EF-hand protein 1 [Source:HGNC Symbol;Acc:HGNC:17433] | 10940 | -0.423 | -0.3686 | Yes |
| 93 | RRS1 | ribosome biogenesis regulator 1 homolog [Source:HGNC Symbol;Acc:HGNC:17083] | 11045 | -0.437 | -0.3664 | Yes |
| 94 | NUP160 | nucleoporin 160 [Source:HGNC Symbol;Acc:HGNC:18017] | 11100 | -0.444 | -0.3602 | Yes |
| 95 | GSK3B | glycogen synthase kinase 3 beta [Source:HGNC Symbol;Acc:HGNC:4617] | 11157 | -0.451 | -0.3539 | Yes |
| 96 | PTPN11 | protein tyrosine phosphatase non-receptor type 11 [Source:HGNC Symbol;Acc:HGNC:9644] | 11260 | -0.465 | -0.3509 | Yes |
| 97 | NUP153 | nucleoporin 153 [Source:HGNC Symbol;Acc:HGNC:8062] | 11307 | -0.472 | -0.3434 | Yes |
| 98 | DDX39A | DExD-box helicase 39A [Source:HGNC Symbol;Acc:HGNC:17821] | 11359 | -0.480 | -0.3361 | Yes |
| 99 | RAPGEF3 | Rap guanine nucleotide exchange factor 3 [Source:HGNC Symbol;Acc:HGNC:16629] | 11380 | -0.483 | -0.3263 | Yes |
| 100 | NCBP3 | nuclear cap binding subunit 3 [Source:HGNC Symbol;Acc:HGNC:24612] | 11502 | -0.501 | -0.3239 | Yes |
| 101 | BAG3 | BAG cochaperone 3 [Source:HGNC Symbol;Acc:HGNC:939] | 11562 | -0.509 | -0.3165 | Yes |
| 102 | TSC1 | TSC complex subunit 1 [Source:HGNC Symbol;Acc:HGNC:12362] | 11575 | -0.511 | -0.3054 | Yes |
| 103 | DESI1 | desumoylating isopeptidase 1 [Source:HGNC Symbol;Acc:HGNC:24577] | 11617 | -0.519 | -0.2964 | Yes |
| 104 | RPS15 | ribosomal protein S15 [Source:HGNC Symbol;Acc:HGNC:10388] | 11671 | -0.527 | -0.2881 | Yes |
| 105 | ZC3H11A | zinc finger CCCH-type containing 11A [Source:HGNC Symbol;Acc:HGNC:29093] | 11780 | -0.546 | -0.2836 | Yes |
| 106 | LSG1 | large 60S subunit nuclear export GTPase 1 [Source:HGNC Symbol;Acc:HGNC:25652] | 11783 | -0.546 | -0.2709 | Yes |
| 107 | PTPN14 | protein tyrosine phosphatase non-receptor type 14 [Source:HGNC Symbol;Acc:HGNC:9647] | 11797 | -0.548 | -0.2590 | Yes |
| 108 | DDX19A | DEAD-box helicase 19A [Source:HGNC Symbol;Acc:HGNC:25628] | 11821 | -0.553 | -0.2478 | Yes |
| 109 | NUP188 | nucleoporin 188 [Source:HGNC Symbol;Acc:HGNC:17859] | 11877 | -0.567 | -0.2387 | Yes |
| 110 | THOC6 | THO complex 6 [Source:HGNC Symbol;Acc:HGNC:28369] | 11918 | -0.576 | -0.2282 | Yes |
| 111 | SSB | small RNA binding exonuclease protection factor La [Source:HGNC Symbol;Acc:HGNC:11316] | 11955 | -0.584 | -0.2173 | Yes |
| 112 | AGFG1 | ArfGAP with FG repeats 1 [Source:HGNC Symbol;Acc:HGNC:5175] | 12035 | -0.602 | -0.2093 | Yes |
| 113 | IL1B | interleukin 1 beta [Source:HGNC Symbol;Acc:HGNC:5992] | 12105 | -0.622 | -0.2000 | Yes |
| 114 | YWHAE | tyrosine 3-monooxygenase/tryptophan 5-monooxygenase activation protein epsilon [Source:HGNC Symbol;Acc:HGNC:12851] | 12137 | -0.628 | -0.1876 | Yes |
| 115 | POM121 | POM121 transmembrane nucleoporin [Source:HGNC Symbol;Acc:HGNC:19702] | 12146 | -0.631 | -0.1734 | Yes |
| 116 | UPF2 | UPF2 regulator of nonsense mediated mRNA decay [Source:HGNC Symbol;Acc:HGNC:17854] | 12150 | -0.631 | -0.1587 | Yes |
| 117 | SMG1 | SMG1 nonsense mediated mRNA decay associated PI3K related kinase [Source:HGNC Symbol;Acc:HGNC:30045] | 12268 | -0.668 | -0.1521 | Yes |
| 118 | SMURF1 | SMAD specific E3 ubiquitin protein ligase 1 [Source:HGNC Symbol;Acc:HGNC:16807] | 12344 | -0.688 | -0.1417 | Yes |
| 119 | HNRNPA1 | heterogeneous nuclear ribonucleoprotein A1 [Source:HGNC Symbol;Acc:HGNC:5031] | 12356 | -0.693 | -0.1262 | Yes |
| 120 | NXF1 | nuclear RNA export factor 1 [Source:HGNC Symbol;Acc:HGNC:8071] | 12410 | -0.710 | -0.1137 | Yes |
| 121 | PABPN1 | poly(A) binding protein nuclear 1 [Source:HGNC Symbol;Acc:HGNC:8565] | 12461 | -0.730 | -0.1003 | Yes |
| 122 | THOC1 | THO complex 1 [Source:HGNC Symbol;Acc:HGNC:19070] | 12503 | -0.749 | -0.0859 | Yes |
| 123 | NPM1 | nucleophosmin 1 [Source:HGNC Symbol;Acc:HGNC:7910] | 12530 | -0.762 | -0.0699 | Yes |
| 124 | NEMF | nuclear export mediator factor [Source:HGNC Symbol;Acc:HGNC:10663] | 12531 | -0.762 | -0.0520 | Yes |
| 125 | AKAP13 | A-kinase anchoring protein 13 [Source:HGNC Symbol;Acc:HGNC:371] | 12572 | -0.782 | -0.0366 | Yes |
| 126 | AKAP8L | A-kinase anchoring protein 8 like [Source:HGNC Symbol;Acc:HGNC:29857] | 12665 | -0.836 | -0.0241 | Yes |
| 127 | THOC2 | THO complex 2 [Source:HGNC Symbol;Acc:HGNC:19073] | 12691 | -0.855 | -0.0059 | Yes |
| 128 | DDX39B | DExD-box helicase 39B [Source:HGNC Symbol;Acc:HGNC:13917] | 12783 | -0.941 | 0.0092 | Yes |
Table: GSEA details [plain text format]

  

Fig 2: GOBP\_NUCLEAR\_EXPORT      
 Blue-Pink O' Gram in the Space of the Analyzed GeneSet

  

Fig 3: GOBP\_NUCLEAR\_EXPORT: Random ES distribution      
 Gene set null distribution of ES for **GOBP\_NUCLEAR\_EXPORT**
